# Supplementary figures and images for: Retinitis pigmentosa-linked mutation in DHX38 modulates its splicing activity
Source: PLoS One. 2022 Apr 6;17(4):e0265742. doi: 10.1371/journal.pone.0265742 (PMC8985939; doi:10.1371/journal.pone.0265742)

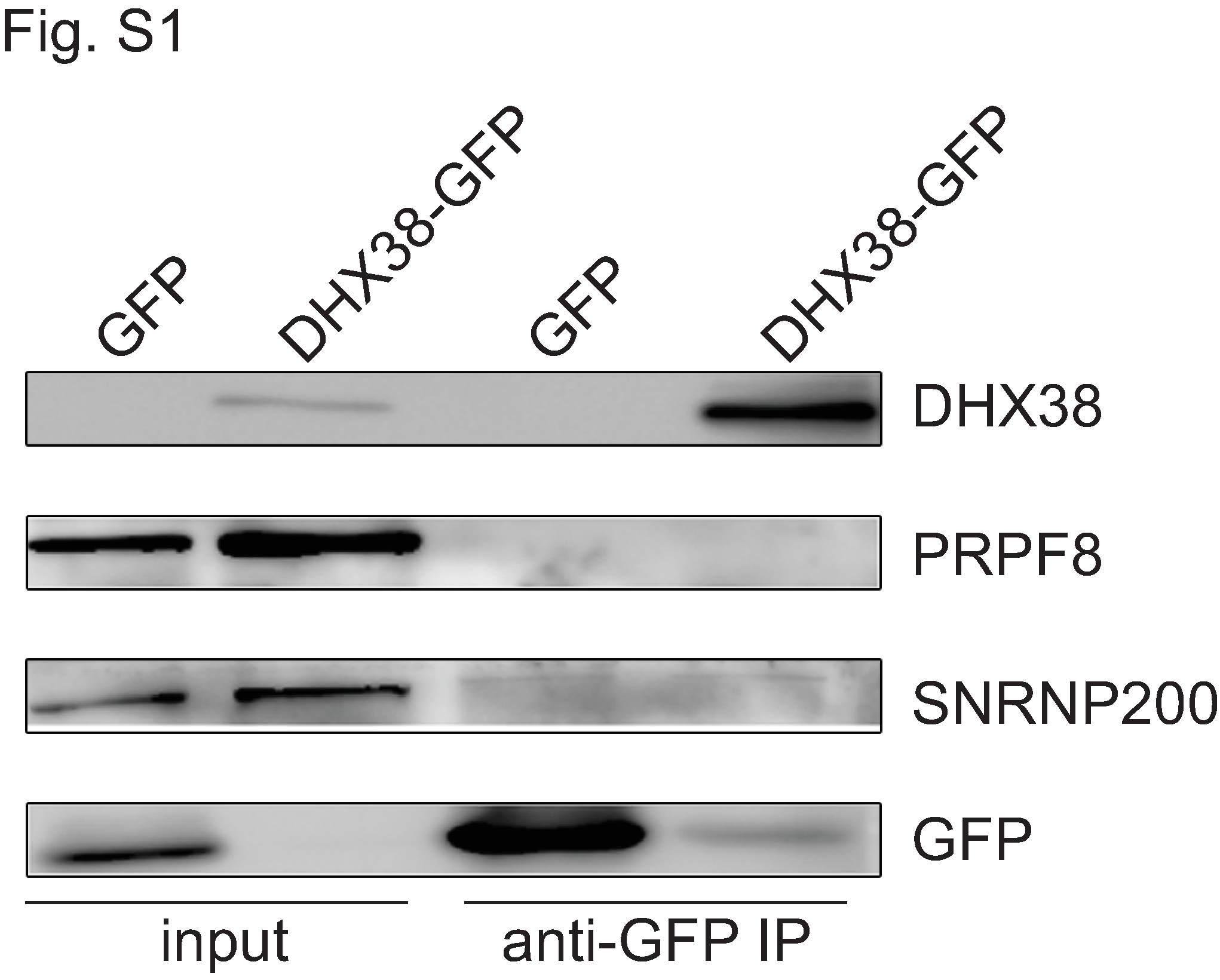

Supplement: S1 Fig — DHX38-GFP was expressed in HEK293 cells, immunoprecipitated and co-purification of PRPF8 and SNRNP200 monitored by Western blotting. (TIF) [file pone.0265742.s001.tif]

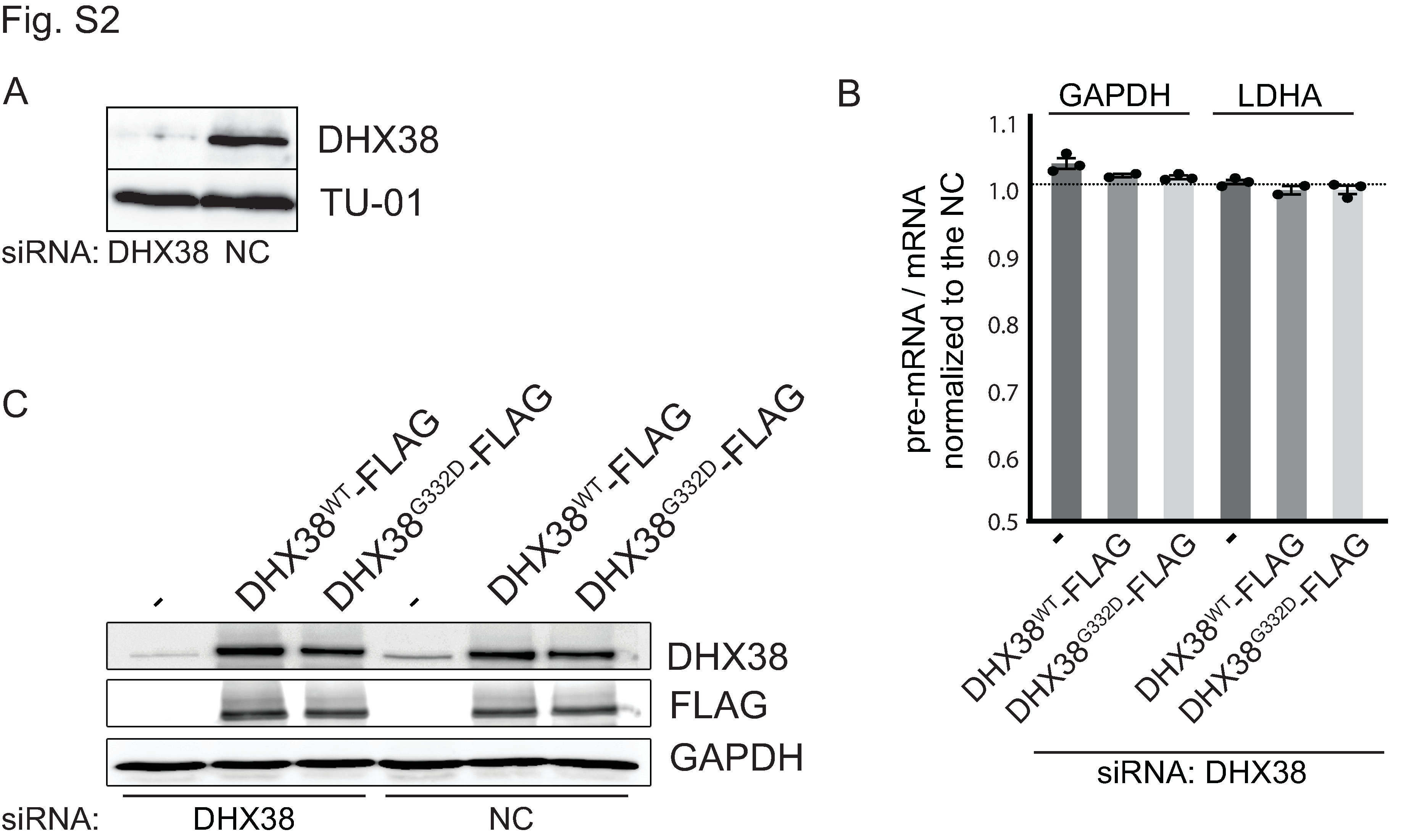

Supplement: S2 Fig — (A) Downregulation of DHX38 by RNAi was monitored by Western blotting. TU-01 (tubulin) served as a loading control (bottom panel). (B) Analysis of LDHA and GAPDH splicing efficiency using RT-qPCR after downregulation of DHX38 and overexpression of DHX38WT-FLAG and DHX38G332D-FLAG proteins. Results of three independent biological experiments are shown together with SEM. (C) siRNA does target siRNA-resistant DHX38WT-FLAG and DHX38G332D-FLAG proteins. Expression of DHX38WT-FLAG and DHX38G332D-FLAG proteins was monitored by Western blotting using the anti-DHX38 antibody (top panel) and the anti-FLAG antibody (middle panel). GAPDH served as a loading control (bottom panel). (TIF) [file pone.0265742.s002.tif]
