# Supplementary material for: Retinitis pigmentosa-linked mutation in DHX38 modulates its splicing activity
Source: PLoS One. 2022 Apr 6;17(4):e0265742. doi: 10.1371/journal.pone.0265742 (PMC8985939; doi:10.1371/journal.pone.0265742)

S1 - File
Raw data for Fig. 1

Raw data for Fig. 1A

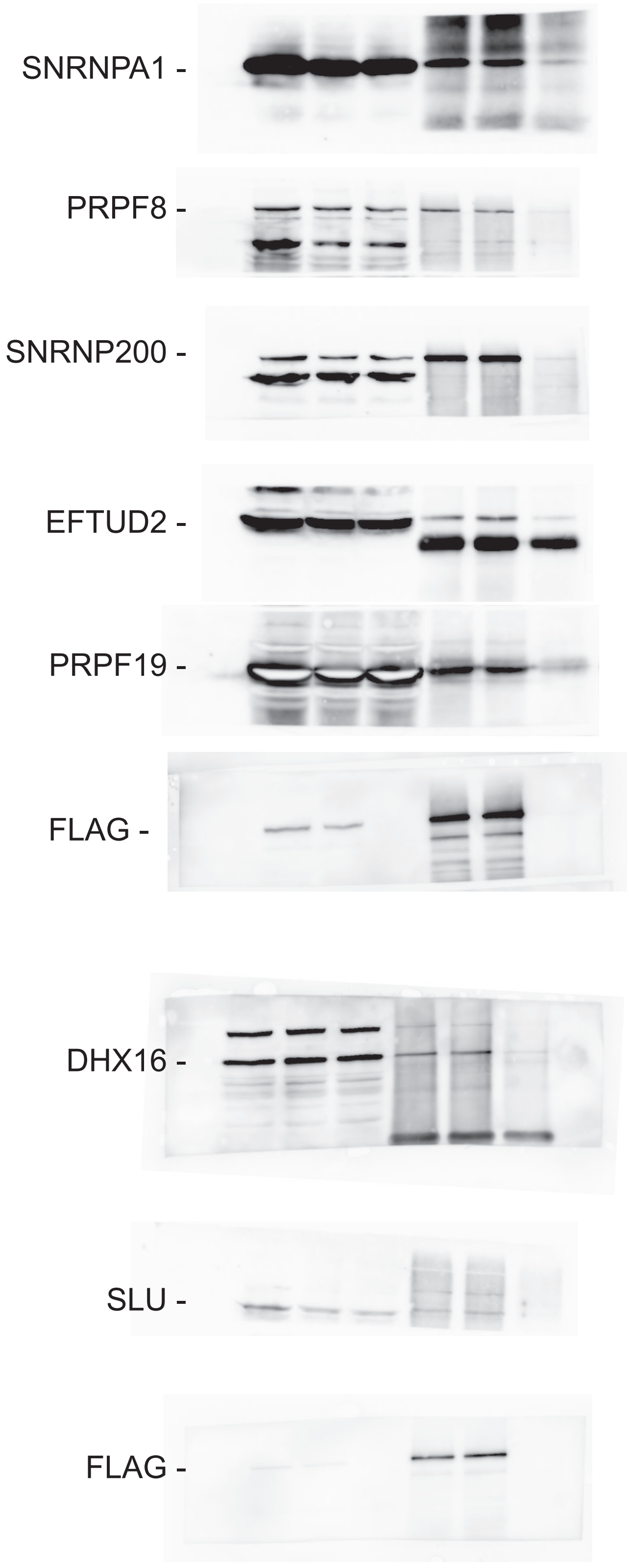

Raw data for Fig. 1B

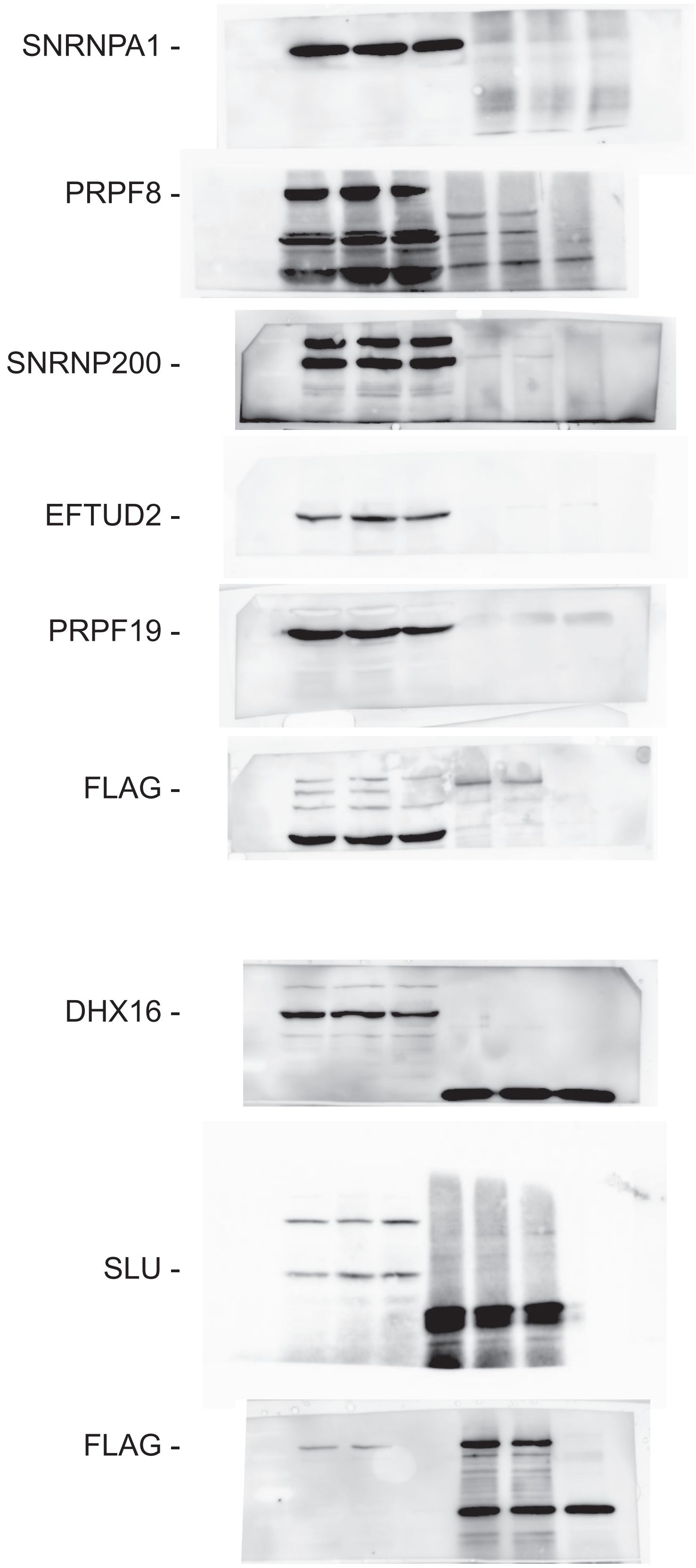

Raw images for Fig 3

Fig. 3A

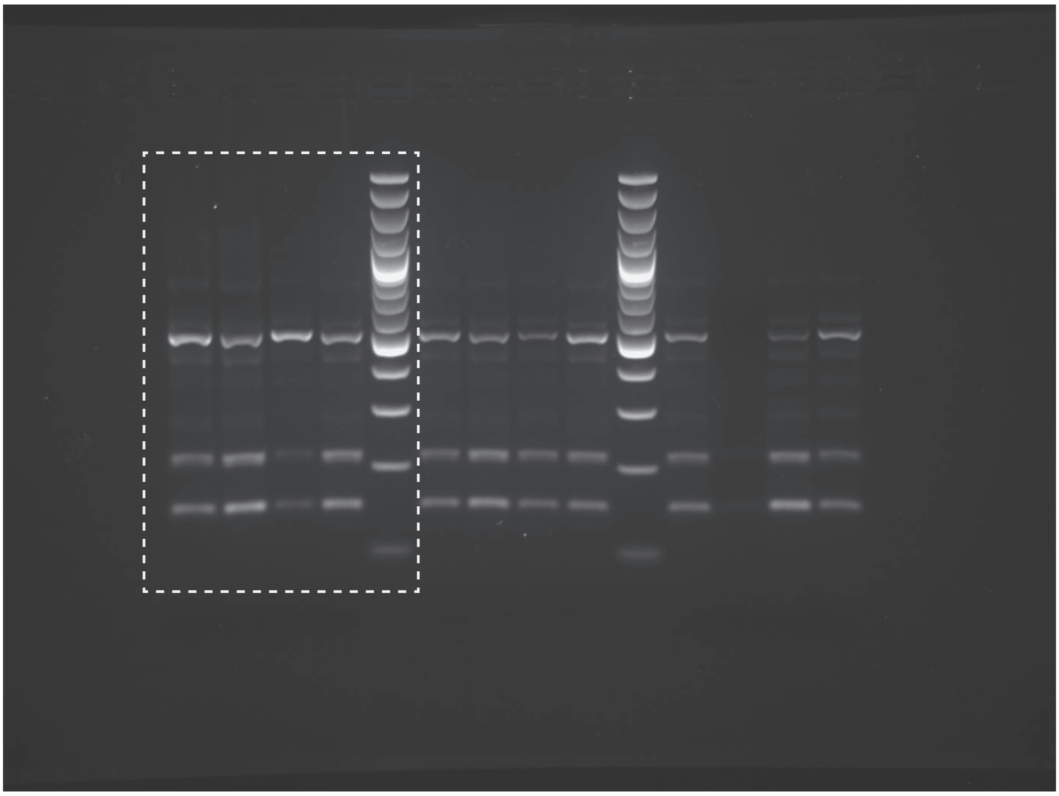

Fig. 3B

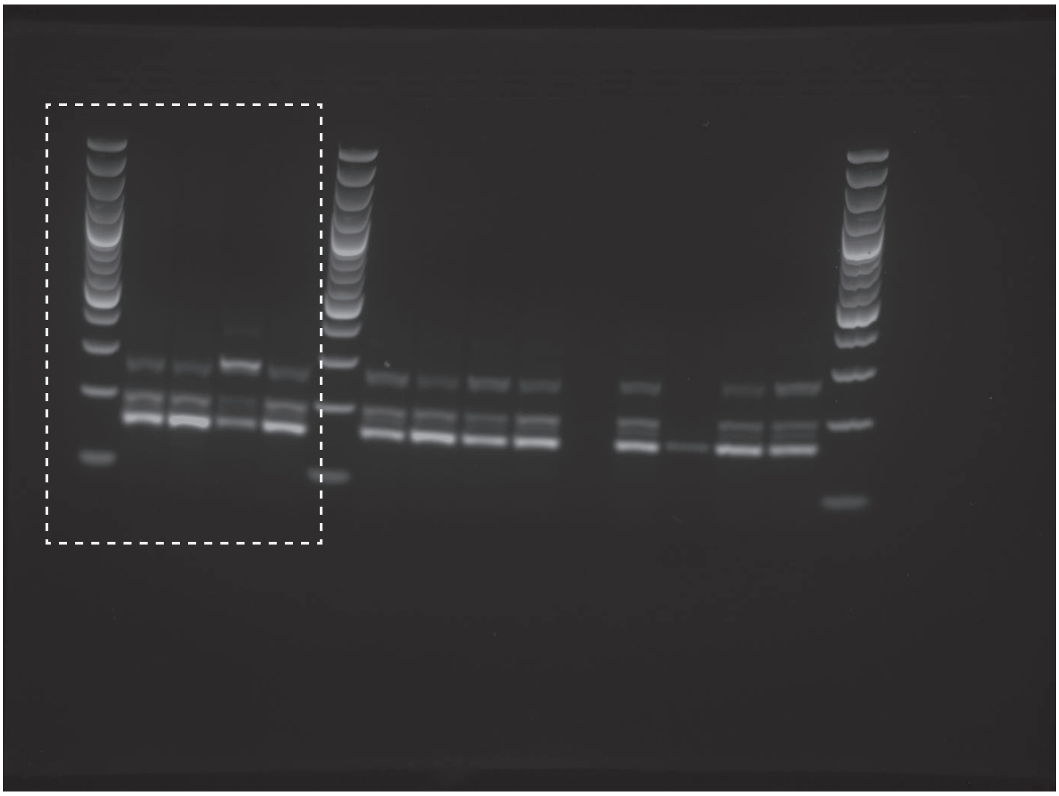

Raw image for Fig. 4A

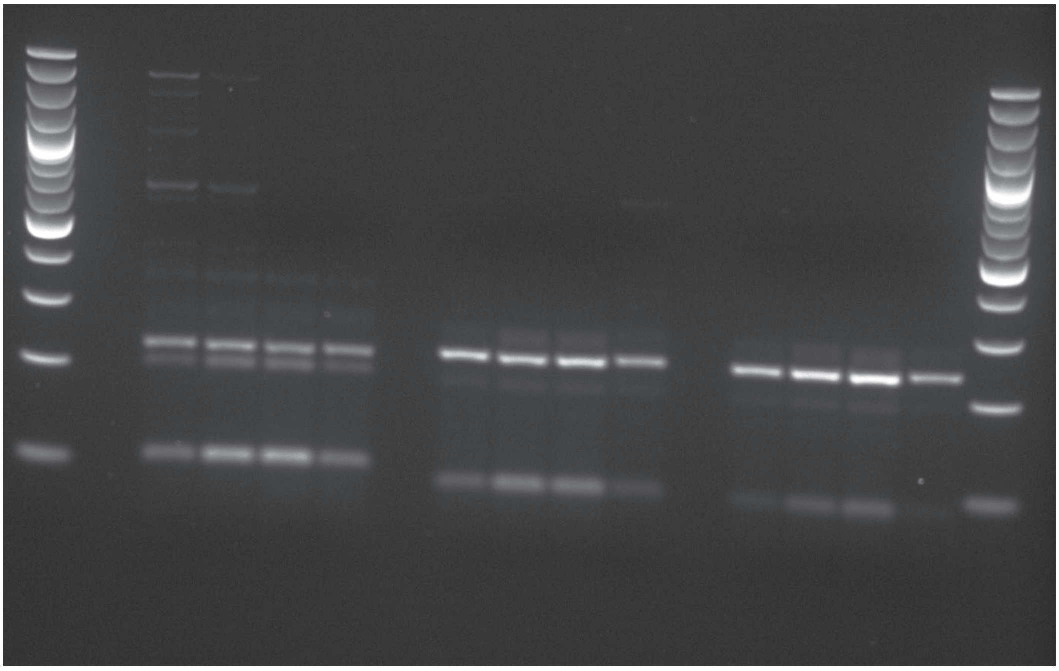

Raw images for Fig. S2C

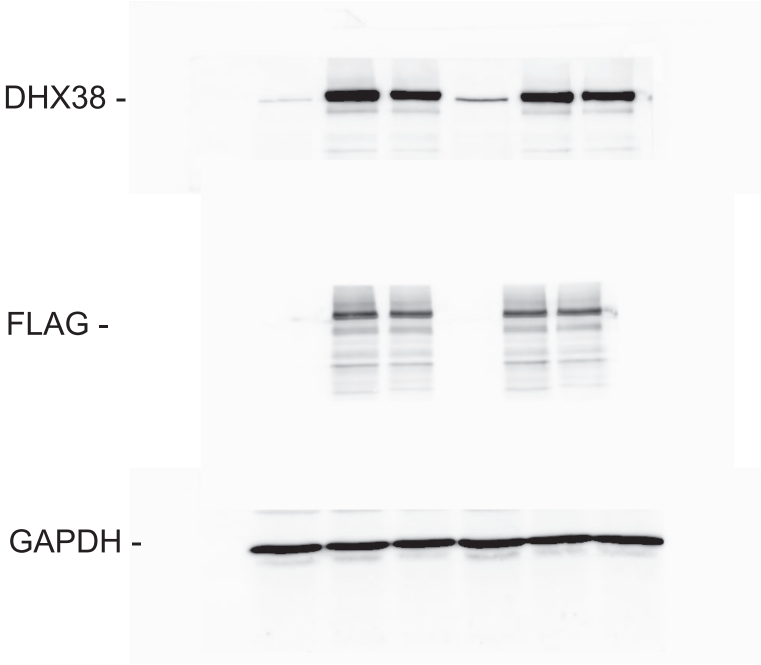

Supplement: S1 Raw images — (PDF) [file pone.0265742.s007.pdf]
