# Supplementary material for: Correction: Retinitis pigmentosa-linked mutation in DHX38 modulates its splicing activity
Source: PLoS One. 2023 Jan 20;18(1):e0280904. doi: 10.1371/journal.pone.0280904 (PMC9858684; doi:10.1371/journal.pone.0280904)
Supplement: S1 File — (PDF) [file pone.0280904.s001.pdf]

PLOS ONE

RESEARCH ARTICLE

Retinitis pigmentosa-linked mutation in DHX38 modulates its splicing activity

Mina Obuća[1,2], Zuzana Cvačková[1], Jan Kubovčiak[1], Michal Kolář[1], David Staněk[1]*

1 Institute of Molecular Genetics of the Czech Academy of Sciences, Prague, Czech Republic, **2** Faculty of Science, Charles University in Prague, Prague, Czech Republic

* stanek@img.cas.cz

Check for updates

OPEN ACCESS

Citation: Obuća M, Cvačková Z, Kubovčiak J, Kolář M, Staněk D (2022) Retinitis pigmentosa-linked mutation in DHX38 modulates its splicing activity. PLoS ONE 17(4): e0265742. https://doi.org/10.1371/journal.pone.0265742

Editor: Alessio Branchini, University of Ferrara: Universita degli Studi di Ferrara, ITALY

Received: November 1, 2021

Accepted: March 7, 2022

Published: April 6, 2022

Copyright: © 2022 Obuća et al. This is an open access article distributed under the terms of the Creative Commons Attribution License, which permits unrestricted use, distribution, and reproduction in any medium, provided the original author and source are credited.

Data Availability Statement: ***PA at Accept: check accession number upon acceptance*** The data underlying the results presented in the study are accessible via ArrayExpress database under accession E-MTAB-11127.

Funding: This work was supported by the Czech Academy of Sciences (RVO68378050 and RVO68378050-KAV-NPUI), the Czech Science Foundation 18-01911J (to Z.C.), the Ministry of Education, Youth and Sports CZ.02.1.01/0.0/0.0/16_019/0000785 (to M.K) and the Charles University Grant Agency 1270217 (to M.O.). The

Abstract

Retinitis pigmentosa (RP) is a hereditary disease affecting tens of thousands of people world-wide. Here we analyzed the effect of an amino acid substitution in the RNA helicase DHX38 (Prp16) causing RP. DHX38 has been proposed as the helicase important for the 2nd step of splicing. We showed that DHX38 associates with key splicing factors involved in both splicing steps but did not find any evidence that the RP mutations changes DHX38 interaction profile with the spliceosome. We further downregulated DHX38 and monitored changes in splicing. We observed only minor perturbations of general splicing but detected modulation of ~70 alternative splicing events. Next, we probed DHX38 function in splicing of retina specific genes and found that FSCN2 splicing is dependent on DHX38. In addition, RHO splicing was inhibited specifically by expression of DHX38 RP variant. Finally, we showed that overexpression of DHX38 promotes usage of canonical as well as cryptic 5' splice sites in HBB splicing reporter. Together, our data show that DHX38 is a splicing factor that promotes splicing of cryptic splice sites and regulate alternative splicing. We further provide evidence that the RP-linked substitution G332D modulates DHX38 splicing activity.

Introduction

Pre-mRNA splicing is a crucial step in gene expression in all eukaryotes when non-coding sequences (introns) are removed and coding sequences (exons) are ligated together. Splicing is executed through a series of tightly coordinated steps. First, the exon-intron boundary is recognized by U1 small nuclear ribonucleoprotein particle (snRNP) and the branch point by the U2 snRNP and its auxiliary factors, all together forming so called A complex. Then, the preassembled U4/U6•U5 tri-snRNP associates with the A complex and through a series of reorganization steps the activated B^{act} complex is formed. The B^{act} complex is transformed into the C complex that executes a two-step splicing reaction. The transformation of individual spliceosomal complexes as well as conformational changes during splicing, release of mRNA and recycling of snRNPs is catalyzed by specific RNA helicases [1]. One of these helicases called Prp16 has been extensively studied in yeast where it was shown to be critical for 2nd step of splicing [2–4]. It was suggested that Prp16 releases Cwc25 and Yju2 proteins during spliceosome activation and remodels the U2-U6 snRNA helix I [4–6]. The *in vitro* study utilizing purified

funder had no role in study design, data collection and analysis, decision to publish, or preparation of the manuscript.

Competing interests: The authors have declared that no competing interests exist.

spliceosomal complexes confirmed Prp16-driven spliceosome rearrangements after the 1st step [7]. Prp16 activity in yeast is regulated by another spliceosomal helicase Brr2 [8]. Prp16 has been also proposed to proofread splicing [9]. Several studies identified Prp16 as a critical factor for recognition of the correct branch-point nucleotide [10–12] and for proofreading of 5' splice-site cleavage [13].

Less is known about Prp16 human homologue DHX38 (also named PRP16, DDX38, KIAA0224). It was identified in human spliceosomal complexes B and C but the signal was much weaker than for example helicase PRP22/DHX8 [14, 15]. DHX38 interaction with the spliceosome is mediated via the G-patch protein GPKOW [16]. Similar to its yeast counterpart, human DHX38 is important for 2nd step of *in vitro* splicing reaction [17, 18].

Retinitis pigmentosa (RP) is a hereditary disease caused by mutations in one hundred genes (sph.uth.edu/retnet/). RP is associated with progressive degeneration of photoreceptors, which eventually triggers migration of retinal pigment epithelium cells to the inner retina, which are visible as dark spots, a hallmark that gave the name to the disease. Most of the targeted genes are specifically expressed in retina but mutations in several ubiquitously expressed splicing factors including DHX38 were associated with retina degeneration as well [19, 20]. Till today, there were identified two recessive mutations upstream of the helicase domain of DHX38 [21, 22]. Mutations are located in a protein domain implied in protein-protein interactions but the effect of RP mutations on DHX38 function is unclear. In general, RP-linked mutations in proteins involved in splicing alter their interaction network and inhibit formation of splicing-competent complexes [23–27]. However, a few RP mutations in PRPF8 and SNRNP200 do not affect assembly of spliceosomal particles but rather change splicing efficiency and fidelity [23, 28].

Here, we analyzed whether and how the RP mutation p.Gly332Asp (G332D) modifies DHX38 interaction with other splicing factors and splicing activity. Using immunoprecipitation under standard and stringent conditions, we monitored co-purification of several splicing proteins with mutated DHX38. We further analyzed an effect of DHX38 downregulation on splicing efficiency and fidelity and tested the ability of mutated DHX38 to rescue the splicing phenotype.

Material and methods

Cloning of DHX38

cDNA of hDHX38 was obtained from Open Biosystems (MHS6278-202828190, clone ID 3504632) and cloned using KpnI and HindIII restriction sites into pEGFP-C2 and using EcoRI and KpnI restriction sites into 3xFLAG vector (pEGFP-C3 vector where EGFP is replaced by 3xFLAG). Point mutation was introduced by site-directed mutagenesis PCR using primers `5′ ATGGACGAGGACTATGACGAG 3′` and `5′ CATGTACCAATCCCGATC 3′`. We inserted the mutation c.G995A, which results in amino acid substitution p.G332D. Constructs were verified by DNA sequencing.

Cell cultivation, siRNA and plasmid transfections, and cycloheximide treatment

HEK293 cells were cultured in high-glucose (4500 mg/l) DMEM medium (Sigma-Aldrich) supplemented with 10% foetal bovine serum, 1% penicillin and streptomycin. Cells were grown in 37˚C and 5% CO_2 atmosphere. To reduce endogenous DHX38, siRNA duplex (`5′ UCAGCUCACAGACCAAAGCGGtt 3′`, Ambion) or siRNA SMART pool (for RNA-seq analysis; targeting sequences: `GCACUGAUCUGGACUGUCA`, `GAUCGGGAUUGGUACAUGA`, `AUGCUAAGGCCAUGCGGAA`, `CCACUCAGCUGACGCAGUA`; ThermoFisher Scientific) were

transfected into cells using Oligofectamine reagent (Invitrogen) at the final concentration of 50nM according to the manufacturer's protocol. The negative control # 5 (NC) siRNA from Ambion was used as a negative control. Cells were incubated for 72h with a change of medium 48h after siRNA transfection. Plasmids were transfected into cells using Lipofectamine LTX (Invitrogen) according to manufacturer's protocol and were analysed 24h after transfection. For splicing assays, we used HBB reporter [28] and retina-specific reporters RHO and FSCN [29, 30] kindly provided by J. Wu (Northwestern University, Chicago, IL). Cycloheximide (CHX) was added to cells at the final concentration 30 ug/ml for 2 hours.

RNA isolation, cDNA synthesis, RT PCR and qPCR

RNA was isolated with TRIzol reagent (Thermo Fisher Scientific) according to manufacturer's protocol. RNA was further precipitated with isopropanol, resuspended in nuclease-free water (Ambion) and treated with Turbo DNase (Ambion) following instructions of the manufacturer. cDNA was synthetized with SuperScript III (Thermo Fisher Scientific) using 500ng of total RNA per 20μl reaction and RT specific primer/random hexamers (Sigma Aldrich). Phusion enzyme was used for PCR reaction in mixture in total 10μl and 2.5μl cDNA. PCR products were separated on 2% agarose gel and the intensity of individual bands was determined using ImageJ software. Quantitative PCR was performed using SYBR Green I Master Mix (Roche) and the LightCycler 480 System (Roche). The reaction volume of 5μl contained 2μl of template cDNA (diluted to 1:10) and 500nM of each primer. Splicing efficiency was calculated as $2^{(Ct[mRNA]-Ct[pre\text{-}mRNA])}$. For RNA seq validation, fold change of differentially expressed genes was calculated as $2^{((Ct[siDHX38,\ gene\ of\ interest]-Ct[siDHX38,\ GAPDH])-(Ct[NC5,\ gene\ of\ interest]-Ct[NC5,\ GAPDH]))}$. Each replica represents an independent time-separated biological experiment. Statistical analysis was performed by two-tailed t-test and the significance was always compared to cells treated with negative control siRNA. The list of used primers is provided in S1 Table.

RNA-seq and data analysis

HEK293 cells from 100 mm in diameter Petri dish were resuspended in 1ml of TRIZOL (Invitrogen) and total RNA was isolated using Direct-zol microprep kit (Zymo research) including DNase I treatment. Good quality RNA (RIN > 9.2) was ribodepleted and used for library preparation. Libraries were prepared using KAPA RNA HyperPrep Kit with RiboErase (Roche) and sequenced on Illumina NextSeq 500 instrument with 2x75 bp configuration. For subsequent data processing, a bioinformatic pipeline nf-core/rnaseq was used (version 1.4.2; Zenodo; https://doi.org/10.5281/zenodo.3503887). Individual steps included removing sequencing adaptors with Trim Galore! (https://www.bioinformatics.babraham.ac.uk/projects/trim_galore/), mapping with HISAT2 [31] and gene expression quantification using Salmon (both based on reference genome GRCh38, Ensembl annotation version 101 [32]). Quantified expression data served as input for differential gene expression analysis using DESeq2 R Bioconductor package [33]. Genes exhibiting |Log2 Fold change|>1 and statistical significance (FDR < 0.05) between compared groups of samples (DHX38 knockdown vs. cells treated with negative control siRNA) were considered as differentially expressed. Furthermore, based on alternative splicing analysis of mapping data with ASpli 2.0 R Bioconductor package [34], genes containing introns exhibiting percentage of intron retained (PIR) difference > 10% were considered as differentially spliced. RNA-seq data are accessible via ArrayExpress database under accession E-MTAB-11127. GO terms gene set overrepresentation analysis of differentially expressed and spliced genes was done using GOSeq R Bioconductor package.

Immunoprecipitation

Plasmids encoding DHX38 WT and mutant proteins were transfected into HEK293 cells using Lipofectamine 3000 (Invitrogen) according to manufacturer's protocol. 24h after the transfection cells were washed 3x with PBS buffer, harvested into PBS and centrifuged at 1 000 g and 4˚C for 10 min. Harvested cells were resuspended in NET2 buffer (50 mM Tris–HCl pH 7.5, 150 mM NaCl or 300 mM NaCl, 0.05% IGEPAL CA-630 (Sigma-Aldrich)) supplemented with RNasin (Promega) and Protease Inhibitor Cocktail Set III (Calbiochem). The samples were sonicated on ice bath (3x30 pulses; 0.5s for each pulse at 60% of maximum energy) and centrifuged at 20,000 g and 4˚C for 10min. Pellets were discarded and 20μl of the supernatant were saved as 'input' samples. The rest of the supernatant was pre-cleaned for subsequent immunoprecipitation by incubating on a rotator with 10μl Protein G Sepharose beads (GE Healthcare) at 4˚C for 2h. Tagged DHX38 was immunoprecipitated by either goat anti-GFP antibodies (kindly provided by Pavel Tomancak and Pavel Mejstrik, Max Planck Institute for Molecular Cell Biology and Genetics, Dresden, Germany) or anti-FLAG M2 antibody (Sigma Aldrich, slbx2256) at 4˚C for 4h.

Western blotting

Proteins were separated by 10% SDS-PAGE and transferred to a Protran 0.45 μm nitrocellulose membrane (GE Healthcare) in transfer buffer (25 mM Tris-HCl, 192 mM glycine, 20% methanol). The membrane was washed with PBST (PBS supplemented with 0.05% Tween) and blocked with 10% low fat milk/PBST. All antibodies were diluted in 1% low fat milk/PBST. AffiniPure goat secondary antibodies conjugated with horseradish peroxidase (Jackson ImmunoResearch) were used. The membrane was incubated with SuperSignal West Femto/Pico Substrate (Thermo Fisher Scientific) and the image was developed using LAS-3000 Imager (Fujifilm).

Antibodies

The following antibodies were used:

mouse anti-DHX38 (Santa Cruz Biotechnology, sc-81081)

rabbit anti-SNRNP200 (Sigma Aldrich, HPA029321)

rabbit anti-U2A (Abcam, ab128937)

mouse anti-PRPF8 (E-5) (Santa Cruz, sc-55533)

rabbit anti-EFTUD2 (kindly provided by Reinhard Lührmann, Max Planck Institute for Biophysical Chemistry, Göttingen, Germany)

mouse anti-PRP19 (Santa Cruz, sc-514338)

mouse anti-DHX16, (Santa Cruz, sc-135879)

mouse anti-SLU7 (B-11) (Santa Cruz, sc-376985)

mouse anti-FLAG M2 (Sigma Aldrich, slbx2256)

goat anti-GFP (kindly provided by Pavel Tomancak and Pavel Mejstrik, Max Planck Institute for Molecular Cell biology and Genetics, Dresden, Germany)

mouse anti-GAPDH (Abcam, AB9484)

mouse anti-GFP (Santa Cruz Biotechnology, sc-9996)

mouse anti-tubulin (TU-01, kindly provided by Pavel Dráber, Institute of Molecular Genetics, Prague, Czech Republic)

Results

To analyze the DHX38 interactome, we cloned human DHX38 into a vector containing the CMV promoter and GFP at the N-terminus of DHX38. However, transiently expressed GFP-DHX38^{WT} did not interact with proteins PRPF8 and SNRNP200, which are key components of B and C spliceosome complexes. This result indicated that the GFP tag might have disrupted DHX38 interaction profile (S1 Fig). We therefore tagged the N-terminus of DHX38 with a smaller FLAG tag, transiently expressed DHX38-FLAG in HEK293 cells and immunoprecipitated with the anti-FLAG antibody. DHX38-FLAG co-precipitated all tested markers of U2 snRNP (SNRNPA1/U2A'), U5 snRNP (PRPF8, SNRNP200, EFTUD2) and the PRPF19 complex (PRPF19). We also detected an association with the step 1 factor DHX16/PRP2 and the step 2 factor SLU7 (Fig 1A). These data indicated that DHX38 is present at various spliceosomal complexes formed during splicing. To test, whether the G332D mutation, which is localized in the putative DHX38 protein-protein interaction domain, disrupts DHX38 association with spliceosome, we inserted the G332D mutation into the DHX38-FLAG construct and compared proteins coprecipitated with wild-type (WT) and mutated DHX38 ^{G332D}-FLAG. However, we did not observe any significant difference between the interaction patterns of DHX38^{WT}-FLAG and DHX38^{G332D}-FLAG proteins (Fig 1A). To probe whether the mutation affects the strength of the interaction with splicing proteins we performed

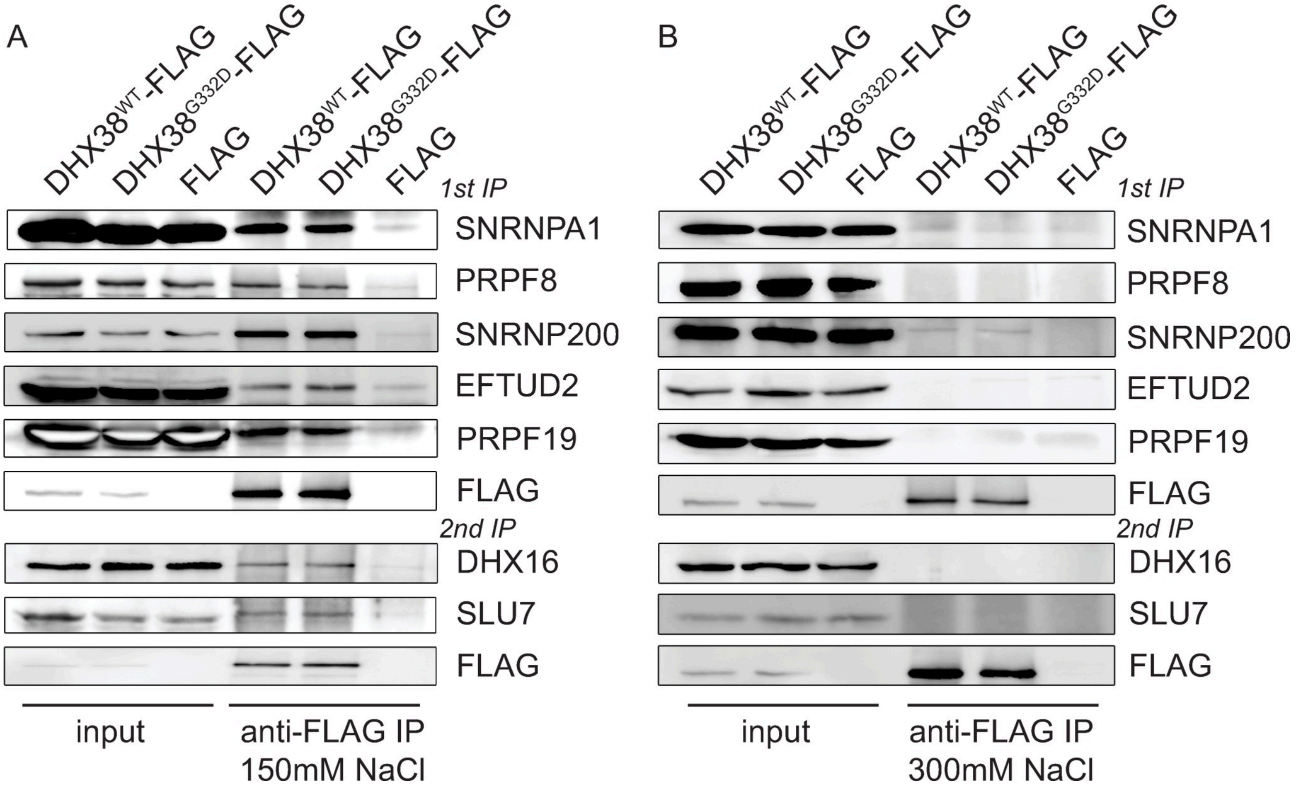

Fig 1. DHX38 associates with multiple spliceosomal components. DHX38^{WT}-FLAG and DHX38^{G332D}-FLAG were transiently expressed in HEK293 cells, immunoprecipitated and copurification of selected spliceosomal proteins monitored. Immunoprecipitation was performed in normal NET2 buffer containing 150 mM NaCl (A) or in stringent NET2 buffer containing 300 mM NaCl (B).

https://doi.org/10.1371/journal.pone.0265742.g001

immunoprecipitation under more stringent salt condition (300 mM NaCl). Almost all interactions of DHX38 were lost in stringent conditions and we detected only a weak signal from the U5 snRNP-specific protein SNRNP200 that co-precipitated with both WT and DHX38^{G332D} proteins (Fig 1B). Because we observed DHX38 interaction with all snRNP markers as well as proteins involved in 1st and 2nd splicing step, we concluded that DHX38 interacts with activated spliceosome during both 1st and 2nd step of splicing and that the G332D mutation does not visibly alter association with splicing complexes.

Next, we tested whether the RP mutation affects pre-mRNA splicing. First, we downregulated DHX38 in HEK293 cells by RNA interference and analyzed cellular RNA by next generation sequencing (RNA-seq). Surprisingly, despite rather efficient DHX38 knockdown (S2A Fig), we observed only a few genes with significant changes in splicing efficiency measured as expression of reads over exon-intron junctions (Fig 2A and S2 Table). Surprisingly, some introns are spliced more efficiently after DHX38 knockdown. We also did not find any defects in splicing efficiency of two house-keeping genes LDHA and GAPDH monitored by classical reverse transcription coupled with quantitative PCR (RT-qPCR), which further indicated that DHX38 downregulation did not inhibit general splicing (S2B Fig). Next, we determined genes where downregulation of DHX38 modified alternative splicing. We specifically focused on exon inclusion/skipping and identified 71 events where exon inclusion was significantly changed after DHX38 downregulation (Fig 2B and S3 Table). To better understand processes after DHX38 downregulation we analyzed genes differentially expressed in cells treated with anti-DHX38 and negative control siRNAs (Fig 2C). We identified 34 genes whose expression significantly (FDR$<$0.05) changed more than twice (S4 Table). We picked 6 upregulated and 6 downregulated genes and probed their expression after DGX38 knockdown by RT-qPCR (Fig 2D and 2E).

GO analysis of differentially spliced and expressed genes revealed that DHX38 knockdown triggered pathways involved in stress response (apoptotic pathways and response to unfolded proteins; Table 1). The comparison of genes with reduced splicing and expression revealed that only one gene (DNA damage inducible transcript 3 (DDIT3)) exhibited reduced splicing and expression, which indicates that changes in gene expression are not driven by differences in their splicing and subsequent degradation by non-sense mediated decay (NMD). To further examine prediction, we treated cells with cycloheximide, a potent NMD inhibitor [35] (Fig 2D and 2E). However, neither of the 6 downregulated genes was upregulated upon the cycloheximide treatment indicating that their reduction is not caused by degradation via the NMD pathway.

These results together suggest that DHX38 is not an essential splicing factor in human cultured cells or that residual protein remained after the RNAi treatment is able to rescue splicing. Instead, our data point to the role of DHX38 in regulation of alternative splicing.

Next, we tested the effect of RP mutation on splicing reporters that were derived from retina-specific genes: Rhodopsin (RHO) and Fascin actin-bundling protein 2 (FSCN2) [29, 30]. We downregulated DHX38 in HEK293 cells and transiently expressed siRNA-resistant DHX38^{WT}-FLAG and DHX38^{G332D}-FLAG proteins (S2C Fig) together with the splicing reporters. Depletion of endogenous DHX38 reduced splicing of FSCN2 gene and expression of either WT or mutated protein rescued FSCN2 reporter splicing and splicing efficiency was partially better than in cells treated with negative control siRNA (Fig 3A). We also sequenced the middle band migrating above the main FSCN2 spliced product. This band represents alternative splicing variant that utilizes the alternative 3' splice site located 72 nucleotides upstream of the major 3' splice site and produces the alternative transcript 2 (https://www.ncbi.nlm.nih.gov/nuccore/NM_001077182.3). Sensitivity of this alternative site to DHX38 expression followed the trend observed for the major splicing variant but the effect was weaker (Fig 3A, var. 2).

Completely different results were observed in RHO splicing. In this case, downregulation of DHX38 did not significantly alter RHO splicing but overexpression of DHX38^{G332D}-FLAG

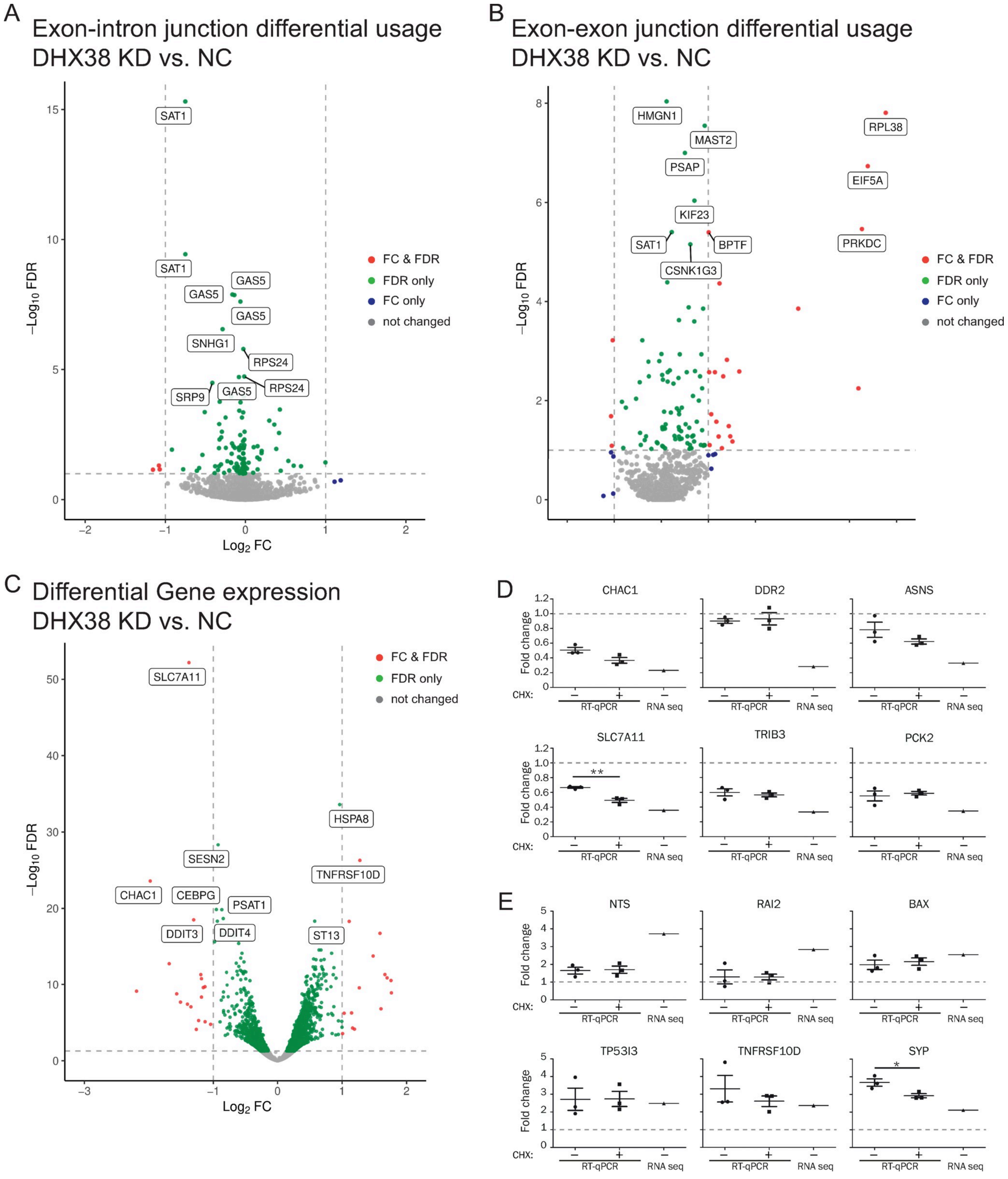

Fig 2. DHX38 downregulation changes primarily gene expression. Summary of three independent RNA-seq analysis of HEK293 cells treated either with anti-DHX38 siRNA or negative control siRNA. Analysis of (A) splicing efficiency, (B) alternative splicing and (C) gene expression. Expression of selected genes that were downregulated (D) or upregulated (E) after DHX38 downregulation was analyzed by RT-qPCR. RNA was isolated from control cells or cells treated with cycloheximide (CHX) to reveal potential targets of non-sense mediated decay. Average of three independent experiment with SEM is shown. Expression value determined by RNA-seq analysis is shown in the same graph for comparison (RNA seq).

https://doi.org/10.1371/journal.pone.0265742.g002

Table 1. GO analysis of differentially spliced and expressed genes after DHX38 knockdown.

**GO analysis of biological processes of differentially expressed genes**			
GO term ID	*GO term*	*Enrichment*	*p-value*
GO:0070059	intrinsic apoptotic signaling pathway in response to endoplasmic reticulum stress	124	1.75e-07
GO:0006986	response to unfolded protein	57.9	6.85e-05
GO:0034620	cellular response to unfolded protein	78.6	7.11e-04
GO:0043525	positive regulation of neuron apoptotic process	62.6	5.46e-05
GO:2001244	positive regulation of intrinsic apoptotic signaling pathway	85.2	1.91e-05
GO:0009636	response to toxic substance	39.8	2.21e-04
GO:0048147	negative regulation of fibroblast proliferation	78.6	7.13e-04
GO:0010506	regulation of autophagy	45.1	1.52e-04
**GO analysis of biological processes of differentially spliced genes**			
GO term ID	*GO term*	*Enrichment*	*p-value*
GO:1903575	cornified envelope assembly	168	4.50e-04
GO:0051090	regulation of DNA-binding transcription factor activity	45	8.82e-04
GO:0031116	positive regulation of microtubule polymerization	43.4	9.79e-04
GO:0006470	protein dephosphorylation	15.6	8.16e-04

https://doi.org/10.1371/journal.pone.0265742.t001

had negative effect on RHO splicing (Fig 3B). Similar to FSCN2 gene, overexpression of WT DHX38-FLAG improved splicing compared to cells treated with negative control siRNA. These data suggested a complex role of DHX38 during splicing and the different sensitivity of specific introns to DHX38 amount. While splicing of FSCN2 depends on DHX38, we did not detect any significant changes in splicing of RHO reporter after the same DHX38 downregulation. In contrast, expression of WT and RP variant rescued FSCN2 splicing but expression of mutated DHX38 significantly inhibited RHO splicing.

Finally, we tested whether RP mutation in DHX38 changes splicing fidelity and monitored usage of cryptic splice sites in a splicing reporter derived from the human hemoglobin beta subunit (HBB) gene [28]. A transiently expressed HBB reporter is not an optimal splicing substrate and approximately half of pre-mRNAs were not spliced (Fig 4A, line 4). However, overexpression of both DHX38^{WT}-FLAG and DHX38^{G332D}-FLAG significantly improved HBB reporter splicing (Fig 4A, lines 2,3). Mutations in 5' splice sites (Fig 4B) forced the splicing machinery to utilize a cryptic 5' splice site located 16 nucleotides upstream of the natural 5' splice site (Fig 4A, lines 5–12). Overexpression of DHX38^{WT}-FLAG stimulated splicing from the cryptic splice site (Fig 4A, lines 6 and 10) and this effect was slightly stronger when mutated DHX38^{G332D}-FLAG was expressed (lines 7 and 11). Quantification of three independent experiments is shown in Fig 4C. Together, these data suggest that overexpression of DHX38 has a positive effect on recognition of weak 5' splice sites and that the RP mutation might strengthen this activity.

Discussion

DEAD/H-box and Ski-like helicases are key executors of conformational changes of the spliceosome that drive the splicing reaction in the right direction. In addition to this essential function, RNA helicases also act as proofreading factors that enhance fidelity of splice site recognition, and proper and timely execution of splicing [1]. Here, we analyzed how an amino acid substitution in the DEAH-box helicase DHX38 (Prp16 in yeast), which causes RP, affects DHX38 function. The mutation is located in the N-terminal half of the protein rich for four amino acids arginine, glutamate, aspartate and serin (REDS). These amino acids comprise

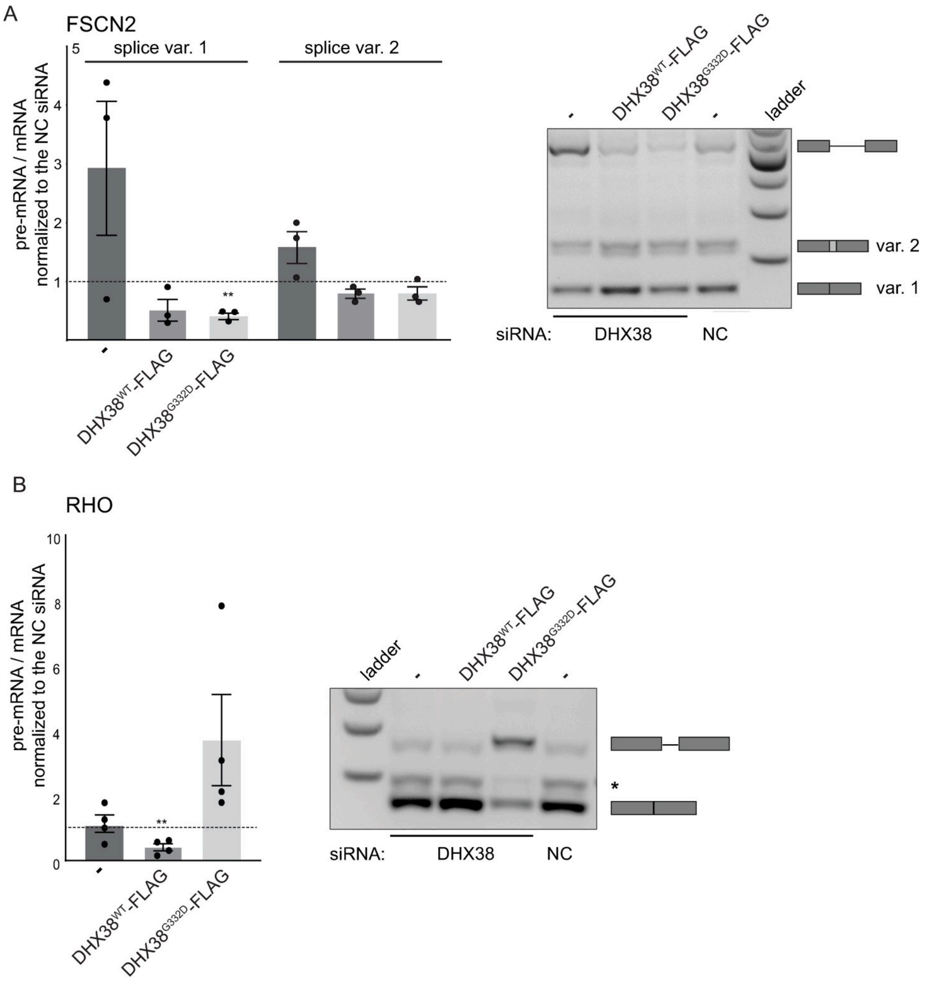

Fig 3. DHX38 is important for splicing of retina gene-derived reporters. Splicing of (A) FSCN2-derived reporter and (B) RHO-derived reporter was analyzed after DHX38 downregulation and after expression of siRNA-resistant DHX38^{WT}-FLAG and DHX38^{G332D}-FLAG proteins. Normalized ratio of pre-mRNA/mRNA from at least three independent experiments is shown in a graph together with the mean and SEM. Statistical significance was assessed by two-tail unpaired t-test (** indicates $p \leq 0.01$). Representative gels are shown. * next to RHO gel marks an unspecific product.

https://doi.org/10.1371/journal.pone.0265742.g003

more than 50% of all amino acids between amino acids 60–400 of DHX38. A similar REDS domain is found in another spliceosomal helicase DHX8/PRP22 and it was speculated that REDS might mediate protein-protein interactions [18]. The mutation changes of neutral Gly to acidic Asp might increase a local negative charge of this protein domain. Here, we mapped interaction partners of DHX38 by immunoprecipitation but did not identify any changes in DHX38-assocItating proteins between WT and RP variant of DHX38 (Fig 1). In addition, we did not observe differential expression of DHX38 protein with the RP mutation (S2 Fig). We concluded that the RP mutation does not influence either the stability of DHX38 or interaction of DHX38 with the spliceosome. However, we cannot exclude that the substitution affects interaction with protein not tested in our assay.

Therefore, we analyzed whether the RP mutation modulates splicing activity of DHX38, which was suggested to be important for the 2nd splicing step. However, reducing DHX38 protein expression by RNAi did not significantly inhibit pre-mRNA splicing and splicing of only a

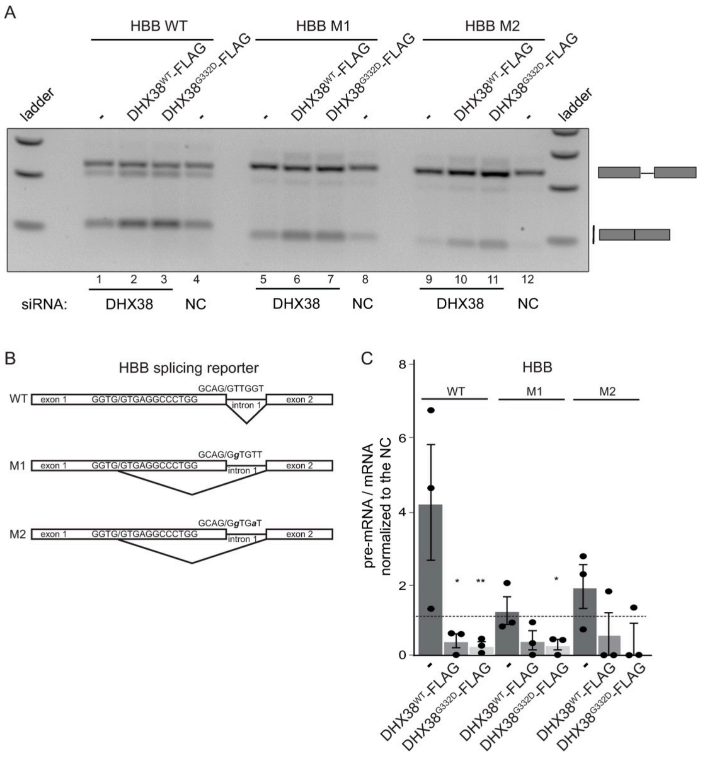

Fig 4. DHX38 promotes splicing of the HBB-derived reporter. (A) A representative gel of HBB-derived reporter splicing after DHX38 knockdown and expression of siRNA-resistant DHX38^{WT}-FLAG and DHX38^{G332D}-FLAG. (B) A scheme of HBB-derived reporter and M1 and M2 mutants that block usage of normal splice site and promote usage of upstream 5' cryptic splice site. (C) Quantification of three independent experiments with the mean and SEM. Statistical significance was assessed by two tail unpaired t-test (* indicates $p \leq 0.05$, ** $p \leq 0.01$).

https://doi.org/10.1371/journal.pone.0265742.g004

handful of genes was significantly affected. We cannot exclude that the remaining amount of DHX38 left after the RNAi treatment support splicing. However, a similar downregulation of a core splicing factor PRPF8 reduced splicing of most of the tested genes [36]. We therefore speculate that DHX38 is not an essential splicing factor but promotes splicing of specific introns. Indeed, splicing efficiency of the FSCN2-derived splicing reporter containing FSCN2 intron 3 was significantly reduced upon DHX38 downregulation (Fig 3). In addition, expression of the RP-variant specifically inhibited splicing of RHO-derived splicing reporter, which indicates that only specific introns are sensitive to changes in DHX38 expression/function. A similar gene specific effects were already observed in case of RP mutations in PRPF31, which specifically inhibited splicing of RHO reporter but not a splicing reporter derived from ROM1 gene [29]. In addition, different RP mutations in PRPF31 had different effect on splicing of various reporter genes [30]. Finally, two RP mutations in SNRNP200 promoted usage of the 5' cryptic splice site in HBB splicing reporter [28]. We observed the same enhancement of splicing from the HBB cryptic spliced site after overexpression of DHX38 and specifically the RP variant of this helicase (Fig 4). Similarly, overexpression of DHX38, both wild-type and RP variant, enhanced splicing of FSCN2 intron (Fig 3). This suggests that DHX38 might promote splicing of weak and suboptimal RNA substrates. A correlation between Prp16-dependent splicing and the strength of the interaction between pre-mRNA and U2 and U6 snRNAs has been previously observed in *Schizosaccharomyces pombe* [37]. Whether this splicing changes also applies to target retina tissues in patients expressing mutated DHX38 requires further investigation using more retina-relevant models.

Conclusions

Our data show that DHX38 is a splicing regulator that primary enhances the splicing of weak and suboptimal introns. RP mutation in DHX38 does not significantly alter its binding profile with other splicing factors but the RP variant of DHX 38 might shift the balance of a splicing regulatory network and sensitive introns and exons could be spliced in partially different ratios than in cells expressing the wild-type DHX38.

Supporting information

S1 Fig. SHX38-GFP does not associate with U5-specific proteins. DHX38-GFP was expressed in HEK293 cells, immunoprecipitated and co-purification of PRPF8 and SNRNP200 monitored by Western blotting.
(TIF)

S2 Fig. Downregulation of DHX38 does not inhibit splicing of selected house-keeping genes. (A) Downregulation of DHX38 by RNAi was monitored by Western blotting. TU-01 (tubulin) served as a loading control (bottom panel). (B) Analysis of LDHA and GAPDH splicing efficiency using RT-qPCR after downregulation of DHX38 and overexpression of $DHX38^{WT}$-FLAG and $DHX38^{G332D}$-FLAG proteins. Results of three independent biological experiments are shown together with SEM. (C) siRNA does target siRNA-resistant $DHX38^{WT}$-FLAG and $DHX38^{G332D}$-FLAG proteins. Expression of $DHX38^{WT}$-FLAG and $DHX38^{G332D}$-FLAG proteins was monitored by Western blotting using the anti-DHX38 antibody (top panel) and the anti-FLAG antibody (middle panel). GAPDH served as a loading control (bottom panel).
(TIF)

S1 Table. List of used primers.
(XLSX)

S2 Table. List of differentially spliced introns.
(XLSX)

S3 Table. List of alternatively spliced exons.
(XLSX)

S4 Table. List of differentially expressed genes.
(XLSX)

S1 Raw images. Raw images for Figs 1, 3, 4 and S2.
(PDF)

Acknowledgments

We also thanks Reinhard Lührmann, Pavel Draber, Pavel Mejstrik and Pavel Tomancak for providing us with antibodies and Jane Wu for plasmids.

Author Contributions

Conceptualization: David Staněk.

Formal analysis: Jan Kubovčiak.

Funding acquisition: Mina Obuća, Zuzana Cvačková, Michal Kolář.

Investigation: Mina Obuća, Zuzana Cvačková.

Project administration: Zuzana Cvačková, David Staněk.

Software: Jan Kubovčiak.

Supervision: Michal Kolář, David Staněk.

Writing – original draft: Zuzana Cvačková, David Staněk.

References

1. Cordin O, Hahn D, Beggs JD. Structure, function and regulation of spliceosomal RNA helicases. Curr Opin Cell Biol. 2012; 24(3):431–8. Epub 2012/04/03. https://doi.org/10.1016/j.ceb.2012.03.004 PMID: 22464735.
2. Schwer B, Guthrie C. A conformational rearrangement in the spliceosome is dependent on PRP16 and ATP hydrolysis. EMBO J. 1992; 11(13):5033–9. Epub 1992/12/01. PMID: 1464325; PubMed Central PMCID: PMC556981.
3. Schwer B, Guthrie C. A dominant negative mutation in a spliceosomal ATPase affects ATP hydrolysis but not binding to the spliceosome. Mol Cell Biol. 1992; 12(8):3540–7. Epub 1992/08/01. https://doi.org/10.1128/mcb.12.8.3540-3547.1992 PMID: 1385854; PubMed Central PMCID: PMC364619.
4. Hogg R, de Almeida RA, Ruckshanthi JP, O'Keefe RT. Remodeling of U2-U6 snRNA helix I during pre-mRNA splicing by Prp16 and the NineTeen Complex protein Cwc2. Nucleic Acids Res. 2014; 42 (12):8008–23. Epub 2014/05/23. https://doi.org/10.1093/nar/gku431 PMID: 24848011.
5. Tseng CK, Liu HL, Cheng SC. DEAH-box ATPase Prp16 has dual roles in remodeling of the spliceosome in catalytic steps. RNA. 2011; 17(1):145–54. Epub 2010/11/26. https://doi.org/10.1261/rna.2459611 PMID: 21098140; PubMed Central PMCID: PMC3004056.
6. Mefford MA, Staley JP. Evidence that U2/U6 helix I promotes both catalytic steps of pre-mRNA splicing and rearranges in between these steps. RNA. 2009; 15(7):1386–97. Epub 2009/05/22. https://doi.org/10.1261/rna.1582609 PMID: 19458033.
7. Ohrt T, Odenwalder P, Dannenberg J, Prior M, Warkocki Z, Schmitzova J, et al. Molecular dissection of step 2 catalysis of yeast pre-mRNA splicing investigated in a purified system. RNA. 2013; 19(7):902–15. Epub 2013/05/21. https://doi.org/10.1261/rna.039024.113 PMID: 23685439; PubMed Central PMCID: PMC3683925.
8. Cordin O, Hahn D, Alexander R, Gautam A, Saveanu C, Barrass JD, et al. Brr2p carboxy-terminal Sec63 domain modulates Prp16 splicing RNA helicase. Nucleic Acids Res. 2014; 42(22):13897–910. Epub 2014/11/28. https://doi.org/10.1093/nar/gku1238 PMID: 25428373; PubMed Central PMCID: PMC4267655.
9. Semlow DR, Staley JP. Staying on message: ensuring fidelity in pre-mRNA splicing. Trends in biochemical sciences. 2012; 37(7):263–73. Epub 2012/05/09. https://doi.org/10.1016/j.tibs.2012.04.001 PMID: 22564363; PubMed Central PMCID: PMC3735133.
10. Burgess SM, Guthrie C. A mechanism to enhance mRNA splicing fidelity: the RNA-dependent ATPase Prp16 governs usage of a discard pathway for aberrant lariat intermediates. Cell. 1993; 73(7):1377–91. doi: 0092-8674(93)90363-U [pii]. https://doi.org/10.1016/0092-8674(93)90363-u PMID: 8324826.
11. Villa T, Guthrie C. The Isy1p component of the NineTeen complex interacts with the ATPase Prp16p to regulate the fidelity of pre-mRNA splicing. Genes Dev. 2005; 19(16):1894–904. Epub 2005/08/17. https://doi.org/10.1101/gad.1336305 PMID: 16103217; PubMed Central PMCID: PMC1186189.
12. Semlow DR, Blanco MR, Walter NG, Staley JP. Spliceosomal DEAH-Box ATPases Remodel Pre-mRNA to Activate Alternative Splice Sites. Cell. 2016; 164(5):985–98. Epub 2016/02/27. https://doi.org/10.1016/j.cell.2016.01.025 PMID: 26919433; PubMed Central PMCID: PMC4979991.
13. Koodathingal P, Novak T, Piccirilli JA, Staley JP. The DEAH box ATPases Prp16 and Prp43 cooperate to proofread 5' splice site cleavage during pre-mRNA splicing. Mol Cell. 2010; 39(3):385–95. Epub 2010/08/14. https://doi.org/10.1016/j.molcel.2010.07.014 PMID: 20705241; PubMed Central PMCID: PMC3722364.
14. Bessonov S, Anokhina M, Will CL, Urlaub H, Luhrmann R. Isolation of an active step I spliceosome and composition of its RNP core. Nature. 2008; 452(7189):846–50. [pii] https://doi.org/10.1038/nature06842 PMID: 18322460.
15. Bessonov S, Anokhina M, Krasauskas A, Golas MM, Sander B, Will CL, et al. Characterization of purified human Bact spliceosomal complexes reveals compositional and morphological changes during spliceosome activation and first step catalysis. RNA. 2010; 16(12):2384–403. Epub 2010/10/29. https://doi.org/10.1261/rna.2456210 PMID: 20980672; PubMed Central PMCID: PMC2995400.
16. Hegele A, Kamburov A, Grossmann A, Sourlis C, Wowro S, Weimann M, et al. Dynamic protein-protein interaction wiring of the human spliceosome. Mol Cell. 2012; 45(4):567–80. Epub 2012/03/01. https://doi.org/10.1016/j.molcel.2011.12.034 PMID: 22365833.

17. Ortlepp D, Laggerbauer B, Mullner S, Achsel T, Kirschbaum B, Luhrmann R. The mammalian homologue of Prp16p is overexpressed in a cell line tolerant to Leflunomide, a new immunoregulatory drug effective against rheumatoid arthritis. RNA. 1998; 4(8):1007–18. Epub 1998/08/13. https://doi.org/10.1017/s1355838298980554 PMID: 9701291; PubMed Central PMCID: PMC1369677.

18. Zhou Z, Reed R. Human homologs of yeast prp16 and prp17 reveal conservation of the mechanism for catalytic step II of pre-mRNA splicing. EMBO J. 1998; 17(7):2095–106. Epub 1998/06/06. https://doi.org/10.1093/emboj/17.7.2095 PMID: 9524131; PubMed Central PMCID: PMC1170554.

19. Ruzickova S, Stanek D. Mutations in spliceosomal proteins and retina degeneration. RNA biology. 2017; 14(5):544–52. https://doi.org/10.1080/15476286.2016.1191735 PMID: 27302685; PubMed Central PMCID: PMC5449078.

20. Krausova M, Stanek D. snRNP proteins in health and disease. Semin Cell Dev Biol. 2018; 79:92–102. Epub 2017/10/19. https://doi.org/10.1016/j.semcdb.2017.10.011 PMID: 29037818.

21. Ajmal M, Khan MI, Neveling K, Khan YM, Azam M, Waheed NK, et al. A missense mutation in the splicing factor gene DHX38 is associated with early-onset retinitis pigmentosa with macular coloboma. Journal of medical genetics. 2014; 51(7):444–8. Epub 2014/04/17. https://doi.org/10.1136/jmedgenet-2014-102316 PMID: 24737827.

22. Latif Z, Chakchouk I, Schrauwen I, Lee K, Santos-Cortez RLP, Abbe I, et al. Confirmation of the Role of DHX38 in the Etiology of Early-Onset Retinitis Pigmentosa. Investigative ophthalmology & visual science. 2018; 59(11):4552–7. Epub 2018/09/13. https://doi.org/10.1167/iovs.18-23849 PMID: 30208423; PubMed Central PMCID: PMC6133250.

23. Malinova A, Cvackova Z, Mateju D, Horejsi Z, Abeza C, Vandermoere F, et al. Assembly of the U5 snRNP component PRPF8 is controlled by the HSP90/R2TP chaperones. J Cell Biol. 2017; 216(6):1579–96. https://doi.org/10.1083/jcb.201701165 PMID: 28515276; PubMed Central PMCID: PMC5461031.

24. Huranova M, Hnilicova J, Fleischer B, Cvackova Z, Stanek D. A mutation linked to retinitis pigmentosa in HPRP31 causes protein instability and impairs its interactions with spliceosomal snRNPs. Hum Mol Genet. 2009; 18(11):2014–23. https://doi.org/10.1093/hmg/ddp125 PubMed PMID: Huranova2009. PMID: 19293337

25. Tanackovic G, Ransijn A, Thibault P, Abou Elela S, Klinck R, Berson EL, et al. PRPF mutations are associated with generalized defects in spliceosome formation and pre-mRNA splicing in patients with retinitis pigmentosa. Hum Mol Genet. 2011; 20(11):2116–30. Epub 2011/03/08. https://doi.org/10.1093/hmg/ddr094 PMID: 21378395; PubMed Central PMCID: PMC3090192.

26. Gonzalez-Santos JM, Cao H, Duan RC, Hu J. Mutation in the splicing factor Hprp3p linked to retinitis pigmentosa impairs interactions within the U4/U6 snRNP complex. Hum Mol Genet. 2008; 17(2):225–39. doi: ddm300 [pii] https://doi.org/10.1093/hmg/ddm300 PMID: 17932117.

27. Linder B, Hirmer A, Gal A, Ruther K, Bolz HJ, Winkler C, et al. Identification of a PRPF4 loss-of-function variant that abrogates U4/U6.U5 tri-snRNP integration and is associated with retinitis pigmentosa. PLoS ONE. 2014; 9(11):e111754. Epub 2014/11/11. https://doi.org/10.1371/journal.pone.0111754 PMID: 25383878; PubMed Central PMCID: PMC4226509.

28. Cvackova Z, Mateju D, Stanek D. Retinitis Pigmentosa Mutations of SNRNP200 Enhance Cryptic Splice-Site Recognition. Human mutation. 2014; 35(3):308–17. Epub 2013/12/05. https://doi.org/10.1002/humu.22481 PMID: 24302620.

29. Yuan L, Kawada M, Havlioglu N, Tang H, Wu JY. Mutations in PRPF31 inhibit pre-mRNA splicing of rhodopsin gene and cause apoptosis of retinal cells. J Neurosci. 2005; 25(3):748–57. https://doi.org/10.1523/JNEUROSCI.2399-04.2005 PMID: 15659613.

30. Mordes D, Yuan L, Xu L, Kawada M, Molday RS, Wu JY. Identification of photoreceptor genes affected by PRPF31 mutations associated with autosomal dominant retinitis pigmentosa. Neurobiology of Disease. 2007; 26(2):291–300. https://doi.org/10.1016/j.nbd.2006.08.026 PubMed PMID: Mordes2007. PMID: 17350276

31. Kim D, Langmead B, Salzberg SL. HISAT: a fast spliced aligner with low memory requirements. Nat Methods. 2015; 12(4):357–60. Epub 2015/03/10. https://doi.org/10.1038/nmeth.3317 PMID: 25751142; PubMed Central PMCID: PMC4655817.

32. Yates AD, Achuthan P, Akanni W, Allen J, Allen J, Alvarez-Jarreta J, et al. Ensembl 2020. Nucleic Acids Res. 2020; 48(D1):D682–D8. Epub 2019/11/07. https://doi.org/10.1093/nar/gkz966 PMID: 31691826; PubMed Central PMCID: PMC7145704.

33. Love MI, Huber W, Anders S. Moderated estimation of fold change and dispersion for RNA-seq data with DESeq2. Genome biology. 2014; 15(12):550. Epub 2014/12/18. https://doi.org/10.1186/s13059-014-0550-8 PMID: 25516281; PubMed Central PMCID: PMC4302049.

34. Mancini E, Rabinovich A, Iserte J, Yanovsky M, Chernomoretz A. Corrigendum to: ASpli: Integrative analysis of splicing landscapes through RNA-Seq assays. Bioinformatics. 2021. Epub 2021/06/11. https://doi.org/10.1093/bioinformatics/btab345 PMID: 34109387.

35. Carter MS, Doskow J, Morris P, Li S, Nhim RP, Sandstedt S, et al. A regulatory mechanism that detects premature nonsense codons in T-cell receptor transcripts in vivo is reversed by protein synthesis inhibitors in vitro. J Biol Chem. 1995; 270(48):28995–9003. Epub 1995/12/01. https://doi.org/10.1074/jbc.270.48.28995 PMID: 7499432.

36. Klimesova K, Vojackova J, Radivojevic N, Vandermoere F, Bertrand E, Verheggen C, et al. TSSC4 is a component of U5 snRNP that promotes tri-snRNP formation. Nat Commun. 2021; 12(1):3646. Epub 2021/06/17. https://doi.org/10.1038/s41467-021-23934-y PMID: 34131137; PubMed Central PMCID: PMC8206348.

37. Vijayakumari D, Sharma AK, Bawa PS, Kumar R, Srinivasan S, Vijayraghavan U. Early splicing functions of fission yeast Prp16 and its unexpected requirement for gene Silencing is governed by intronic features. RNA biology. 2019; 16(6):754–69. Epub 2019/02/28. https://doi.org/10.1080/15476286.2019.1585737 PMID: 30810475; PubMed Central PMCID: PMC6546409.
